# Clinicopathological and Prognostic Characteristics in Dedifferentiated/Poorly Differentiated Chordomas: A Pooled Analysis of Individual Patient Data From 58 Studies and Comparison With Conventional Chordomas

**DOI:** 10.3389/fonc.2021.686565

**Published:** 2021-08-13

**Authors:** Fu-Sheng Liu, Bo-Wen Zheng, Tao-Lan Zhang, Jing Li, Guo-Hua Lv, Yi-Guo Yan, Wei Huang, Ming-Xiang Zou

**Affiliations:** ^1^Health Management Center, The First Affiliated Hospital, University of South China, Hengyang, China; ^2^Department of Spine Surgery, The Second Xiangya Hospital, Central South University, Changsha, China; ^3^Department of Radiation Oncology, Indiana University School of Medicine, IU Simon Comprehensive Cancer Center, Indianapolis, IN, United States; ^4^Department of Spine Surgery, The First Affiliated Hospital, University of South China, Hengyang, China

**Keywords:** dedifferentiated chordoma, poorly differentiated chordoma, comparative study, clinicopathological characteristics, survival analysis, prognostic factors

## Abstract

**Background:**

Currently, the clinicopathological and prognostic characteristics of dedifferentiated chordoma (DC) and poorly differentiated chordoma (PDC) remain poorly understood. In this study, we sought to characterize clinicopathological parameters in a large PDC/DC cohort and determine their correlations with progression-free survival (PFS) and overall survival (OS) of patients. We also attempted to compare clinical features between PDC/DC and conventional chordoma (CC).

**Methods:**

Literature searches (from inception to June 01, 2020) using Medline, Embase, Google Scholar and Wanfang databases were conducted to identify eligible studies according to predefined criteria. The local database at our center was also retrospectively reviewed to include CC patients for comparative analysis.

**Results:**

Fifty-eight studies from the literature and 90 CC patients from our local institute were identified; in total, 54 PDC patients and 96 DC patients were analyzed. Overall, PDC or DC had distinct characteristics from CC, while PDC and DC shared similar clinical features. Adjuvant radiotherapy and chemotherapy were associated with both PFS and OS in PDC patients in the univariate and/or multivariate analyses. In the DC cohort, tumor resection type, adjuvant chemotherapy and tumor dedifferentiation components significantly affected PFS, whereas none of them were predictive of outcome in the multivariate analysis. By analyzing OS, we found that surgery, resection type and the time to dedifferentiation predicted the survival of DC patients; however, only surgery remained significant after adjusting for other covariables.

**Conclusions:**

These data may offer useful information to better understand the clinical characteristics of PDC/DC and may be helpful in improving the outcome prediction of patients.

## Introduction

Chordoma is a rare and locally invasive malignant mesenchymal tumor that is considered to originate from embryonic residual notochordal tissues (1, 2). Chordoma accounts for approximately 1-4% of all bone tumors (3) and has a population-based incidence rate of 0.8/1000000 (4). The peak age for chordoma onset ranges from 60 to 70 years (5–8). Chordomas most commonly involve the sacrococcygeal or skull base (9), and cases occurring outside the axial skeleton have also been reported (10). Chordoma is unresponsive to traditional radiotherapy and chemotherapy; therefore, surgery constitutes the main treatment of choice. Due to its infiltrative nature and proximity to key neurovascular structures, complete resection of chordoma lesions may be challenging (11, 12). Therefore, the risk of tumor recurrence after surgery is high, and 40-60% of patients may even have distant metastasis during the course of disease, exerting a significant adverse effect on the quality of life and survival of patients (1, 13).

Histologically, chordomas can be divided into three subtypes: classical, chondroid and dedifferentiated types (14). Specifically, conventional chordoma (CC) is microscopically characterized by the presence of physaliferous cells in the mucous matrix, while chondroid chordoma contains both chordoma and chondroma components. By contrast, dedifferentiated chordoma (DC) is determined when sarcomatous elements emerge within CC tissues (14). Recently, a new chordoma subtype called poorly differentiated chordoma (PDC) has been proposed based on its clinicopathological and/or genetic features (6). Unlike other subtypes, PDC has no physaliferous cells or rich myxoid matrix on microscopy, although obvious atypia and active mitosis are commonly observed. In addition, it has also been suggested that Brachyury expression is positive, while the expression of SMARCB1/INI-1 is commonly absent in PDC tissues (6, 14, 15).

Currently, reports on the clinical characteristics of and prognostic factors for CC have been widely documented in the literature (16–18). Furthermore, studies have also shown that the prognosis of chondroid chordoma is better than that of CC (19). However, due to the rarity, studies addressing PDC/DC are limited in the literature, with most of them being single case reports or small sample case series. Therefore, little is known about the detailed clinicopathological features of PDC/DC, and no summary of the complete prognostic factors in these two chordoma subtypes has been reported. Previous studies have shown that compared with CC, PDC/DC are highly aggressive with worse patient survival (20–22). Considering the dismal prognosis of PDC/DC, systematically summarizing their prognostic factors may be helpful in stratifying patients and guiding therapeutic optimization, thereby improving patient survival. In this study, we sought to characterize the clinicopathological data of a large PDC/DC cohort and explore factors affecting the progression-free survival (PFS) and overall survival (OS) of patients. We also attempted to analyze the differences in clinicopathological and prognostic features between PDC/DC and CC.

## Methods and Materials

### Literature Review

**Figure 1:**
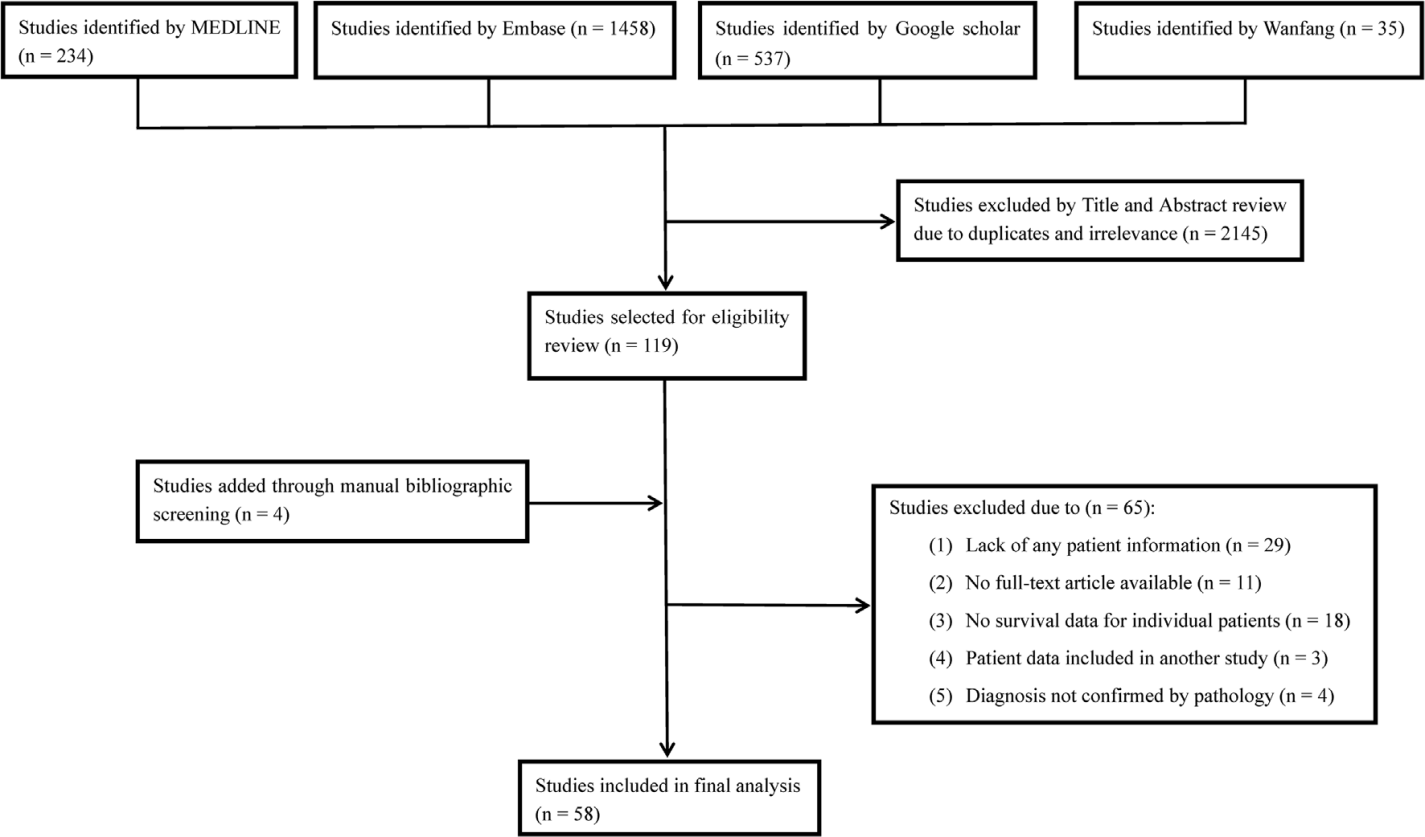
Flow diagram of literature search showing studies identified, included and excluded at each stage.

Electronic searches of MEDLINE, Embase, Google Scholar and Wanfang databases were performed from inception to June 1, 2020, to identify eligible studies. The keywords used for searching were (“chordoma” or “chordomas”) and (“poorly differentiated” or “anaplastic” or “dedifferentiated” or “dedifferentiation” or “sarcomatous” or “sarcomatoid” or “transformation” or “malignant fibrous histiocytoma”). No other restrictions were put on the above searching terms in order to obtain comprehensive outcomes and avoid omissions. Bibliographies of the included studies were also manually checked to find any additional eligible records. The detailed search strategy is depicted in **Figure 1**. We included studies that assessed PDC/DC cases originally occurring in the spine or the skull base areas. Tumor diagnosis was confirmed by pathological and immuno- histochemical findings. Studies were restricted to English or Chinese reports involving human beings. The study exclusion criteria were as follows: no clinical information recorded for patients, duplicate publications, unconfirmed diagnosis of PDC/DC, and failure to provide survival data.

Two investigators independently selected eligible studies according to predefined criteria and extracted data from each included study. These data included the following: patient information (age, sex, duration of symptoms), tumor features [including location, size, dedifferentiation type (*de novo* or secondary), treatment history before tumor dedifferentiation 9such as surgery or radiotherapy), dedifferentiation components and time to dedifferentiation], immunohistochemical results of proteins expression (including Brachyury, pancytokeratin [CK], S-100, vimentin and epithelial membrane antigen [EMA]), treatment (including surgery or not, the type of resection, adjuvant radiotherapy and chemotherapy), endpoint events (tumor progression and death) and time to critical events. The type of resection was determined as wide (such as gross total or *en bloc* resection with negative margins) or not wide (including intralesional or marginal resection) according to a previously described method (11). The main data of interest were PFS and OS. PFS was defined as the time interval from the date of diagnosis to the earliest date of disease progression (local and distant) or death from all causes if no progression was observed (16). Similarly, OS was defined as the interval from the date of diagnosis to death related to all causes or to the date of last follow-up if the patient was alive or lost to follow-up. For subsequent statistical analysis, immunohistochemical results were simply classified into two subgroups according to a published method (10): the high expression group (defined when the protein was diffusely or strongly positive in tumor tissues) and the low expression group (determined when the protein was weakly or focally positive and negative in tumor tissues). For patients with DC, the time to dedifferentiation was defined as the period from initial diagnosis of CC to the first appearance of dedifferentiated sarcomatous component in tumor tissues by pathological examinations.

### Local Cohort

A retrospective review of our pathological database was conducted to identify patients at our center. A total of 90 patients with CC who underwent radical surgery at our institute from June 2002 to November 2018 were included for a comparative analysis with PDC or DC cohort. However, no PDC or DC cases were identified in our institute. Seventy-five of the CC patients had been previously described in our study (10). All patients received regular clinical and radiological examinations after surgery, and the follow-up information was updated in April 2019. The tumor diagnosis, pathological classification and resection type were evaluated by two experienced neuropathologists based on the findings in HE-staining sections of intraoperatively resected specimens. Expression levels of proteins in 75 patients were directly obtained from our published study (10), and the data of 15 newly added patients were retrieved by immunohistochemical analysis. To ensure comparability, the same antibodies (**Supplemental Digital Content 1**), dilution ratio and staining procedures were used during the experiment. Protein expression data were separated into two groups (low and high expression) according to the criteria described above. Patient demographics, treatment history, clinical outcomes and tumor features were obtained from medical records. Observations were censored when a patient was progression-free (PFS analysis) or alive (OS analysis).

### Statistical Analysis

All statistical analyses were performed using R version 3.6.2 (R Foundation for Statistical Computing, Vienna, Austria) and the “surv_cutpoint” function in the “survminer” package was used to determine cut-off values for patient age (in PDC cohort), as well as duration of symptoms and time to tumor dedifferentiation (both in DC data) in survival analysis with OS as the outcome parameter (**Supplemental Digital Content 2**) (23). Specifically, the threshold value was defined as the point with the minimum P value in the log-rank test, which was subsequently corrected to avoid overstating the significance (24). For analysis, the data of patient age in other two cohorts (≤ 50 yr or > 50 yr) and tumor size in all cohorts (≤ 5 cm or > 5 cm), as well as duration of symptoms in CC cohort (≤ 36.5 mo or > 36.5 mo) were separated into two groups as previously suggested (10). Continuous data are expressed as the mean ± standard deviation and were analyzed by Student’s t-test or one-way ANOVA. Categorical data are summarized as the frequency or composition ratio and were analyzed by chi-square test. Univariate survival analysis was performed by Kaplan-Meier curves using the log-rank test to compare survival probabilities between groups. Multivariate survival analysis was conducted with a Cox proportional hazards model to identify independent predictive factors of PFS and OS. This analysis included variables that were statistically significant in our univariate analysis as well as the prognostic factors reported in the literature (14, 21). All tests were two-sided, and a *P* < 0.05 was considered statistically significant.

## Results

### Patient and Tumor Features of the PDC, DC, and CC Cohorts

A total of 58 studies (**Supplemental Digital Content 3**) were finally included, resulting in 150 PDC/DC cases for analysis, including 54 PDC cases and 96 DC cases. For comparative analysis, 90 CC cases were also included in the study. The patient characteristics are detailed in **Table 1**. Most of the included studies did not offer complete clinicopathological data for patients.

**Table 1:** Comparison of baseline characteristics among patients with classic chordoma (CC, n = 90), dedifferentiated chordoma (DC, n = 96) and poorly differentiated chordoma (PDC, n = 54).

Variable	Categories	PDC (n)	DC (n)	CC (n)	*P*-value (PDC *vs.* CC)	*P*-value (DC *vs.* CC)	*P*-value (PDC *vs.* DC)
Age (years)	Continuous	54 (10.35 ± 11.02)	96 (50.34 ± 22.66)	90 (55.02 ± 15.01)	<**0.001**	0.101	< **0.001**
Gender	Female	33	36	61	0.471	<**0.001**	**0.006**
	Male	21	60	29			
Duration of symptoms (months)	Continuous	14 (4.61 ± 5.14)	41 (18.71 ± 29.56)	90 (24.13 ± 26.27)	**0.007**	0.295	0.083
Location	Skull base	33	20	13	<**0.001**	0.337	<**0.001**
	Spine	21	76	77			
Tumor size (largest diameter, cm)	Continuous	21 (5.32 ± 2.41)	44 (10.79 ± 4.57)	90 (6.53 ± 4.27)	0.213	<**0.001**	<**0.001**
Type of resection	Not wide	14	46	37	**0.003**	**0.002**	0.252
	Wide	3	23	53			
Adjuvant chemotherapy	No	23	59	–	–	–	0.118
	Yes	19	26	–			
Adjuvant radiotherapy	No	14	31	67	<**0.001**	<**0.001**	1.000
	Yes	29	64	23			
Brachyury expression	Low	5	1	42	<**0.001**	**0.007**	1.000
	High	43	12	48			
S-100 expression	Low	27	6	67	0.403	<**0.001**	<**0.001**
	High	14	28	23			
CK expression	Low	1	1	66	<**0.001**	<**0.001**	1.000
	High	50	37	24			
EMA expression	Low	1	3	68	<**0.001**	<**0.001**	0.612
	High	25	25	22			
Vimentin expression	Low	0	2	69	NA^a^	<**0.001**	NA^a^
	High	7	21	21			
INI-1 expression	Negative	50	4	0	<**0.001**	NA^a^	NA^a^
	Positive	1	3	90			
Dead	No	27	27	55	0.848	<**0.001**	**0.001**
	Yes	15	60	35			
Progression	No	13	19	31	1.000	0.180	0.271
	Yes	24	58	59			
PFS (months)	Continuous	25 (15.28 ± 17.13)	58 (14.72 ± 14.66)	90 (21.63 ± 17.21)	0.105	**0.013**	0.879
OS (months)	Continuous	40 (22.46 ± 17.46)	76 (23.02 ± 22.35)	90 (40.32 ± 36.20)	**0.004**	<**0.001**	0.890

Bold values indicate P < 0.05; CK, pancytokeratin; EMA, epithelial membrane antigen; INI-1, integrase interactor 1; PFS, progression-free survival; OS, overall survival; ^a^statistical analyses were not performed due to insufficient data.

#### PDC Cohort

In the PDC cohort, 39 patients (72.2%) underwent surgery, and of them, 3 (5.6%) had wide resection, 14 (25.9%) had nonwide resection, and the remaining 22 had an unknown type of surgical resection. Nineteen patients received chemotherapy (13 were treated with unknown chemotherapy regimens; four, with tazemetostat; one, with doxorubicin, vincristine and ifosfamide; and one, with tazemetostat, nivolumab and ipilumimab), while 23 patients did not receive chemotherapy. Twenty-nine patients received adjuvant radiotherapy (12 were treated with unknown radiotherapy types; 5, with pencil beam scanning; 3, with conventional photon radiotherapy; 2, with 3D conformal radiation therapy; and 7, with combinatorial adjuvant radiotherapy), and 14 patients did not. The median PFS was 17.0 months, and the 1-year, 3-year, and 5-year PFS rates were 56%, 27%, and 14%, respectively. The median OS was 52.0 months, and the 1-, 3- and 5-year OS rates were 80%, 51%, and 34%, respectively.

#### DC Cohort

In the DC cohort, 29 patients had treatment history before tumor dedifferentiation (9 patients underwent tumor surgical resection, 8 received adjuvant radiotherapy, and 12 had both tumor resection and radiotherapy), and 67 patients did not. The average time to tumor dedifferentiation was 5.74 ± 6.53 years. Fifty-eight DC cases had dedifferentiation component data available. Of them, 18 tumors had fibrous sarcomatous components (including 11 with malignant fibrous histiocytomas, 5 with fibrosarcomas and 2 with both types), and 40 harbored nonfibrous sarcomatous elements (including 4 with osteosarcomas, 1 with rhabdomyoblastoma, 2 with rhabdomyosarcomas and 33 with other sarcomas). Twenty-three patients (24.0%) underwent wide resection, and 46 patients (47.9%) had nonwide resection. Twenty-six patients (27.1%) received chemotherapy (6 were treated with unknown chemotherapy drugs; two, with doxorubicin; one, with etoposide; and seventeen, with combinatorial adjuvant chemotherapy), and 59 patients did not. Sixty-four patients (66.7%) received adjuvant radiotherapy (including 20 treated with unknown radiotherapy approaches; 26, with traditional photon radiotherapy; 9, with proton beam radiation; 5, with external beam radiotherapy; 3, with carbon-ion radiotherapy; and one, with intensity-modulated radiotherapy), while 31 patients did not receive radiotherapy. The median PFS was 11.7 months, and the 1-, 3- and 5-year PFS rates were 48%, 17%, and 7%, respectively. The median OS was 26.0 months, and the OS rates at 1 year, 3 years and 5 years were 59%, 43% and 19%, respectively.

#### CC Cohort

Clinicopathological data of 90 CC patients have been partially communicated by our group (10, 23). No patients received chemotherapy. All patients underwent surgery. Of them, 53 (58.9%) underwent wide resection, and 37 (41.1%) had nonwide resection. Twenty-three patients (25.6%) received adjuvant radiotherapy (including 13 treated with traditional photon radiotherapy and 10 treated with intensity-modulated radiotherapy), and 67 patients (74.4%) did not. The median PFS was 22.0 months, and the 1-, 3- and 5-year PFS rates were 73%, 20% and 14%, respectively. The median OS was 73.0 months, and the OS rates at 3 years, 5 years and 10 years were 60%, 51% and 39%, respectively.

### Comparison of Clinicopathological Characteristics Among Patients With Different Chordoma Subtypes

The comparative results of clinicopathological features between PDC, DC and CC patients are shown in **Table 1**. Overall, we found significantly different clinicopathological characteristics between the PDC or DC and CC groups, while PDC and DC displayed overlap in their clinical data. Of note, however, as no CC patients received adjuvant chemotherapy, we therefore did not analyze chemotherapy data in this group. In addition, our analysis revealed that the PDC and DC cohorts had more patients undergoing adjuvant radiotherapy than the CC group. However, this outcome might be biased by the fact that the CC cohort included only small number of patients with skull base chordoma who were more likely to be treated with radiotherapy after surgery.

Immunohistochemical results showed that protein biomarkers were differentially expressed among the three chordoma subtypes (**Table 1**). Specifically, INI-1 protein was negatively expressed in most PDC tissues, while all CC cases had positive INI-1 expression (**Supplemental Digital Content 4**), suggesting that INI-1 is a reliable maker for PDC diagnosis. However, due to the lack of enough INI-1 expression information in DC, we therefore did not assess these data in this group. This was also the case for tumoral vimentin expression in the PDC cohort.

**Figure 2:**
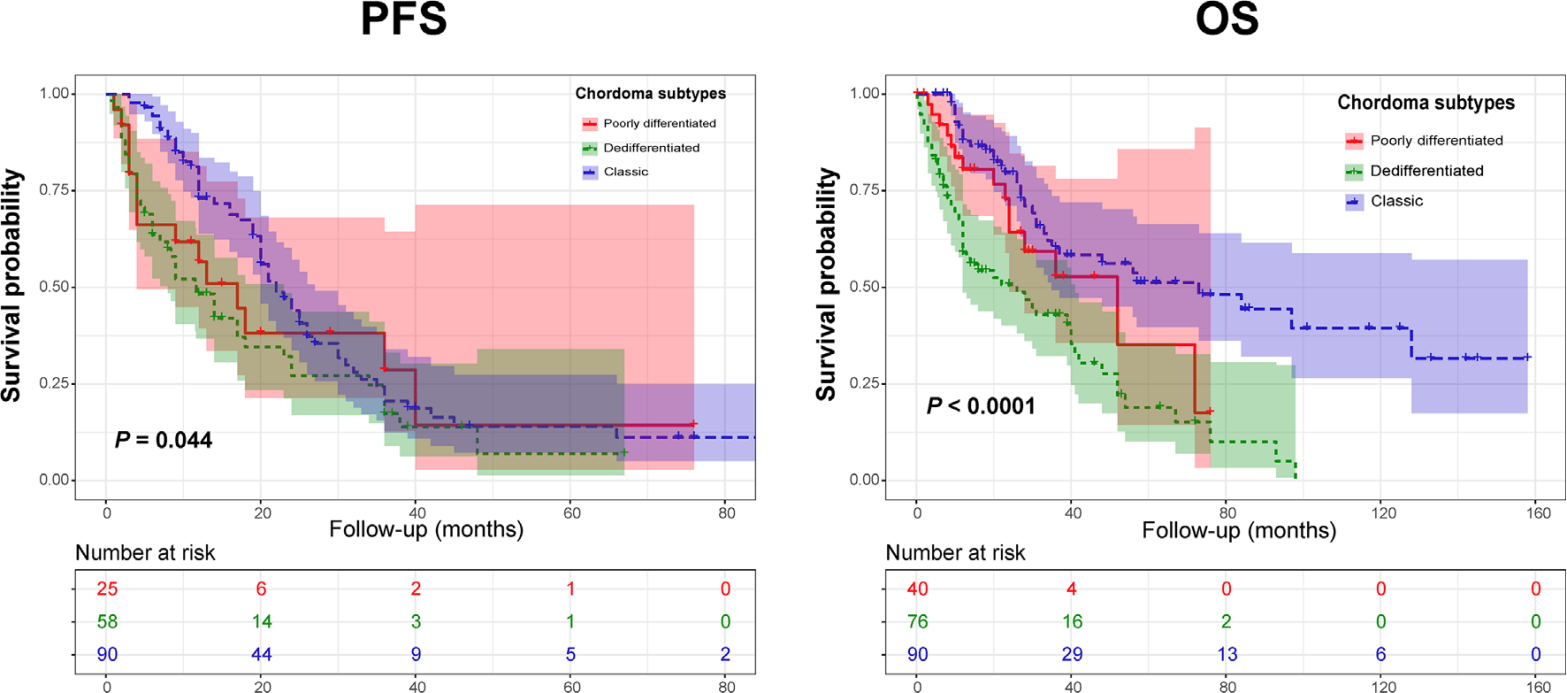
Kaplan-Meier curves of progression-free survival (*Left*) and overall survival (*Right*) of chordoma patients stratified by tumor pathology subtypes.

Regarding clinical outcomes, we found that the PFS of DC patients was significantly shorter than that of CC patients. In addition, a significantly worse OS was observed in the PDC and DC groups than in the CC cohort. Similarly, Kaplan-Meier curves by the log-rank test also revealed significant differences in terms of PFS and OS among the three chordoma subtypes (**Figure 2**). Further subgroup analysis revealed that there was a remarkable difference in PFS between the DC and CC groups (median PFS: 11.7 *vs* 22.0 mo, *P* = 0.012). Similarly, a significant difference was also seen in the OS between the DC and CC groups (median OS: 26.0 *vs* 73.0 mo, *P* < 0.001), but there was only a borderline significant survival difference between the PDC and DC groups (median OS: 52.0 *vs* 26.0 mo, *P* = 0.062).

### Univariate and Multivariate Analyses of Prognostic Factors in PDC and DC Patients

#### PDC Cohort

**Figure 3:**
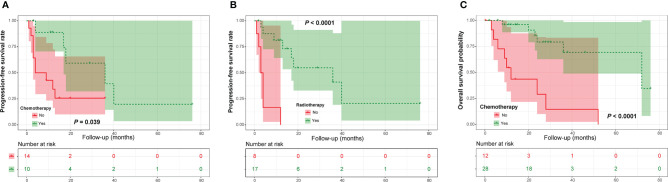
Kaplan-Meier curves of progression-free survival of patients with poorly differentiated chordoma stratified by adjuvant chemotherapy **(A)** and radiotherapy **(B)**, as well as overall survival of patients stratified by adjuvant chemotherapy **(C)**.

In the PDC cohort, univariate Kaplan-Meier analysis showed that adjuvant radiotherapy and chemotherapy were closely related to a good PFS (**Figures 3A, B** and **Table 2**). Similarly, our analysis found that adjuvant radiotherapy was positively associated with OS (**Figure 3C** and **Table 2**). In addition, our results also revealed a marginally significant effect of adjuvant chemotherapy on patients’ OS (χ^2^ = 3.221, *P* = 0.073) (**Table 2**). Multivariate Cox regression analysis showed that adjuvant radiotherapy and chemotherapy could independently predict a good PFS (**Table 3**), while only radiotherapy was an independent predictor of OS (**Table 3**).

**Table 2:** Univariate analyses of prognostic factors for progression-free survival and overall survival in patients with poorly differentiated chordoma^a^.

Factors	Categories	Progression-free Survival				Overall Survival			
		n	Median Survival (months)	χ^2^	*P*-value	n	Median Survival (months)	χ^2^	*P*-value
Age (years)	Young (≤ Cutoff)^b^	16	17.00	0.320	0.571^c^	27	36.00	2.887	0.089^c^
	Old (> Cutoff)^b^	9	13.00			13	–		
Gender	Female	15	17.00	0.361	0.548	26	52.00	0.150	0.699
	Male	10	36.00			14	36.00		
Tumor size (largest diameter, cm)	≤ 5	5	13.00	0.864	0.353	8	–	0.096	0.757
	> 5	8	40.00			10	52.00		
Tumor location	Skull base	15	12.00	2.222	0.136	23	36.00	2.18	0.140
	Spine	10	36.00			17	52.00		
Surgical treatment	No	4	18.00	0.750	0.387	5	–	0.032	0.858
	Yes	20	12.00			34	52.00		
Type of resection	Not wide	5	4.00	0.018	0.892	10	24.00	0.552	0.458
	Wide	1	36.00			2	28.00		
Adjuvant chemotherapy	No	14	4.00	4.241	**0.039**	20	23.00	3.221	0.073
	Yes	10	36.00			19	–		
Adjuvant radiotherapy	No	8	3.00	16.481	<**0.001**	12	12.00	16.082	<**0.001**
	Yes	17	36.00			28	72.00		
Brachyury expression	Low	3	–	2.875	0.090	4	–	2.121	0.145
	High	19	–			32	–		
CK expression	Low	1	36.00	0.003	0.995	1	–	0.651	0.420
	High	22	17.00			37	–		
S-100 expression	Low	15	18.00	0.007	0.932	22	36.00	0.001	0.973
	High	6	13.00			9	28.00		

Bold values indicate P < 0.05; CK, pancytokeratin; INI-1, integrase interactor 1; ^a^analyses were not performed for the association between patient outcomes and duration of symptoms, as well as expression of epithelial membrane antigen, INI-1 and vimentin on tumor cells due to insufficient data; ^b^Cutoff point for patient age in survival analysis was 12; ^c^P-value from the log-rank test was corrected as previously suggested.

**Table 3:** Multivariate Cox proportional hazard analyses of prognostic factors for progression-free survival and overall survival in patients with poorly differentiated chordoma.

Factors	Categories	Progression-free Survival		Factors	Categories	Overall Survival	
		*P*-value	HR (95% CI)			*P*-value	HR (95% CI)
Adjuvant chemotherapy	No/Yes	0.400	1.783 (0.452-7.030)	Adjuvant Chemotherapy	No/Yes	0.321	1.798 (0.565-5.722)
Adjuvant radiotherapy	No/Yes	**0.007**	8.010 (1.762-36.410)	Adjuvant Radiotherapy	No/Yes	**0.002**	6.223 (1.985-19.507)

Bold values indicate P < 0.05.

#### DC Cohort

**Figure 4:**
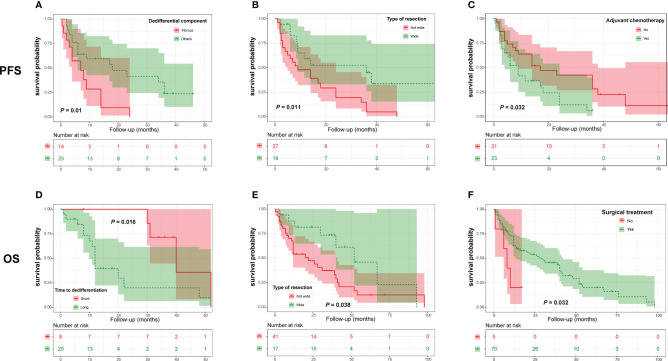
Kaplan-Meier curves of progression-free survival of patients with dedifferentiated chordoma stratified by dedifferentiation component **(A)**, resection type **(B)** and adjuvant chemotherapy **(C)**, as well as overall survival of patients stratified by time to tumor dedifferentiation **(D)**, type of resection **(E)** and surgery **(F)**.

In the DC group, we found that a nonfibrous dedifferentiated component and wide tumor resection were associated with a good PFS (**Figures 4A, B** and **Table 4**). However, in contrast to PDC, adjuvant chemotherapy negatively influenced the PFS of DC patients (**Figure 4C** and **Table 4**). By analyzing OS, we found that the time to tumor dedifferentiation, surgery and resection type were correlated with OS (**Figures 4D–F** and **Table 4**). Multivariate Cox analysis showed that only wide resection of tumors seemed to have a significant correlation with patients’ PFS (*P* = 0.098, **Table 5**). Similarly, this analysis only disclosed that surgical treatment was an independent predictor of OS (**Table 5**).

**Table 4:** Univariate analyses of prognostic factors for progression-free survival and overall survival in patients with dedifferentiated chordoma^a^.

Factors	Categories	Progression-free Survival				Overall Survival			
		*n*	Median Survival (months)	χ^2^	*P*-value	n	Median Survival (months)	χ^2^	*P*-value
Age (years)	≤ 50	20	9.00	0.585	0.444	43	20.00	2.366	0.124
	> 50	29	17.00			33	40.00		
Gender	Female	22	14.00	0.041	0.840	29	30.00	0.003	0.954
	Male	36	6.00			47	22.00		
Type of dedifferentiation	*De novo*	42	14.00	0.011	0.915	48	26.00	0.476	0.490
	Secondary	16	9.00			28	22.00		
Treatment before dedifferentiation	Surgery or RT	10	8.00	0.574	0.449	16	30.00	0.047	0.829
	both	6	9.00			12	20.00		
Time to dedifferentiation (years)	Short (≤ Cutoff)^b^	5	14.00	1.230	0.267^c^	8	40.00	5.831	**0.016** ^c^
	Long (> Cutoff)^b^	11	8.00			20	12.00		
Duration of symptoms (months)	Short (≤ Cutoff)^b^	29	14.00	0.002	0.960^c^	32	30.00	0.683	0.409^c^
	Long (> Cutoff)^b^	4	4.00			8	6.00		
Tumor size (largest diameter, cm)	≤ 5	4	9.00	0.060	0.806	5	54.00	0.096	0.757
	> 5	31	14.00			36	48.00		
Tumor location	Skull base	8	9.00	0.560	0.454	17	28.00	0.060	0.806
	Spine	50	14.00			59	26.00		
Dedifferential component	Fibrous	14	7.00	6.627	**0.010**	16	12.00	1.201	0.273
	Others	25	18.00			35	31.00		
Surgical treatment	No	4	11.67	0.152	0.696	5	8.00	4.709	**0.030**
	Yes	54	11.53			70	28.00		
Type of resection	Not wide	27	9.00	6.461	**0.011**	41	22.00	4.310	**0.038**
	Wide	18	36.00			17	52.00		
Adjuvant chemotherapy	No	31	17.00	4.583	**0.032**	51	20.00	0.128	0.721
	Yes	23	9.00			25	26.00		
Adjuvant radiotherapy	No	15	7.00	0.074	0.786	20	28.00	0.172	0.678
	Yes	43	14.00			56	26.00		
Brachyury expression	Low	1	–	0.343	0.558	1	–	0.434	0.510
	High	7	–			8	–		
EMA expression	Low	2	6.00	0.001	0.970	3	12.00	0.720	0.396
	High	16	14.00			20	22.00		
CK expression	Low	1	–	0.874	0.350	1	–	0.724	0.395
	High	20	–			31	–		
S-100	Low	3	6.00	1.288	0.256	5	12.00	0.355	0.551
	High	15	14.00			23	14.00		

Bold values indicate P < 0.05; CK, pancytokeratin; EMA, epithelial membrane antigen; INI-1, integrase interactor 1; RT, radiotherapy; ^a^analyses were not performed for the association between patient outcomes and tumoral expression of INI-1 and vimentin due to insufficient data; ^b^Cutoff points for time to dedifferentiation and duration of symptoms in the survival analysis were 1.17 and 24, respectively; ^c^P-value from the log-rank test was corrected as previously suggested.

**Table 5:** Multivariate Cox proportional hazard analyses of prognostic factors for progression-free survival and overall survival in patients with dedifferentiated chordoma.

Factors	Categories	Progression-free Survival		Factors	Categories	Overall Survival^a^		Overall Survival^a^	
		*P*-value	HR (95% CI)			*P*-value	HR (95% CI)	*P*-value	HR (95% CI)
Adjuvant Chemotherapy	Yes/No	0.415	1.464 (0.586-3.654)	Time to Dedifferentiation	> Cutoff/≤ Cutoff	0.067	3.182 (0.922-10.975)	0.200	2.831 (0.576-13.911)
Surgical treatment	Yes/No	0.098	0.378 (0.119-1.195)	Surgical treatment	Yes/No	**0.014**	0.095 (0.015-0.622)		
Dedifferential component	Others/Fibrous	0.281	0.582 (0.218-1.557)	Tumor location	Skull base/Spine	0.596	1.349 (0.446-4.074)	0.234	2.241 (0.593-8.470)
				Type of resection	Wide/Not wide			0.283	0.342 (0.048-2.423)

Bold values indicate P < 0.05; ^a^surgical treatment and resection type were not simultaneously included in multivariate analysis of overall survival because they are closely correlated and seem to be confounded with each other.

### Univariate and Multivariate Analyses of Prognostic Factors in CC Patients

Prognostic factors in the CC group have been previously reported in our study (10, 23). After updating the sample size in the study, we found that gender, the duration of symptoms, tumor size, the type of resection, radiotherapy and tumoral Brachyury expression were significantly correlated with PFS (**Supplemental Digital Contents 5-6**). Moreover, this analysis revealed that the duration of symptoms, type of tumor resection and Brachyury expression significantly affected patients’ OS (**Supplemental Digital Contents 6-7**). Further Cox multivariate analysis showed that gender, the type of tumor resection, adjuvant radiotherapy and Brachyury expression were independent prognostic factors of PFS (**Supplemental Digital Content 8**), while the duration of symptoms, type of resection and Brachyury expression independently predicted OS (**Supplemental Digital Content 8**).

## Discussion

In this study, we characterized the clinicopathological data and prognostic factors in a large PDC/DC cohort and compared their features with those of a CC cohort. We found that there were significantly distinct clinical features between PDC/DC and CC, while PDC and DC showed similarities except for patient age, gender, tumor location, size and S-100 expression. In the PDC group, adjuvant radiotherapy and chemotherapy were closely associated with patient outcomes. For DC patients, the type of tumor resection, adjuvant chemotherapy and tumor dedifferentiation components significantly affected PFS, while surgery, the type of resection and time to dedifferentiation predicted OS. These data may contribute to a comprehensive understanding of the clinicopathological and prognostic characteristics of PDC/DC and may be helpful in guiding prognostic risk stratification of and therapeutic optimization for patients.

Consistent with previous studies claiming that PDC and DC are aggressive and have a poor prognosis (6, 14, 17, 25, 26), we found that the prognosis of PDC/DC was poorer than that of CC, with DC patients having the shortest survival. These results may suggest different tumorigenesis mechanisms among PDC/DC and CC, thereby leading to different disease phenotypes and clinical outcomes. Given that PDC is more common in children, we speculate that the occurrence of PDC may be attributed to abnormal molecular mechanisms related to growth and development, which deserves further investigation. As DC can occur *de novo* or after previous treatments, future studies using single-cell sequencing by collecting tissue samples before and after tumor dedifferentiation or matched DC and CC specimens may be helpful in dissecting the precise molecular mechanism of DC development. Such studies are imperative, as they may provide insight into the development of new treatment strategies for DC, considering that the prognosis of this disease is dismal and that the current treatment is highly limited. Furthermore, these therapeutic choices may also be beneficial for PDC patients because PDC and DC shared marked similarity in clinical features as revealed by our data. Noticeably, however, we found no significant survival difference between patients with PDC and CC, in contrast to previous works (14, 20). This phenomenon may be caused by the small number of PDC patients with complete survival data, thus resulting in low statistical power.

Previous studies have confirmed that the type of tumor resection is the main factor affecting the clinical outcomes of chordoma patients (27–29). In contrast, however, we found no significant correlation between the type of resection and patient survival in PDC, which may be due to the small sample size in the survival analysis. It has been shown that PDC (especially for INI-1-negative cases) displays aggressive behavior and tends to have early distant metastasis (20, 30). These conditions may distort evaluation of the true effect of resection type on PDC outcomes. Another major finding of the study was that chemotherapy could improve the survival of PDC patients, similar to previous data (14). One possible reason may be that PDC has poor extracellular matrix; therefore, drugs can easily enter tumor cells to exert their antitumor effects. A recent study provided evidence in support of this conjecture (31). Published data have demonstrated that administration of radiotherapy has benefits in chordoma patients (32–34). Similarly, we found that radiotherapy significantly improved PDC prognosis and could also independently predict both patients’ PFS and OS. These findings together suggest that adjuvant radiotherapy should be performed whenever possible to enhance the survival of PDC patients. Nevertheless, it should be noted that as most of the included studies did not provide detailed information on radiotherapy, we were unable to evaluate the association between specific radiotherapy types and patient outcomes in PDC.

It has been described that dedifferentiated components in DC tissues may include malignant fibrous histiocytoma (35), fibrosarcoma (36), osteosarcoma (37), rhabdomyoblastoma (38) and rhabdomyosarcoma (39). Our study discovered that the presence of a nonfibrous dedifferentiation element had significantly positive prognostic implications in DC, which was in line with previous reports (40). Prior data have suggested that cancer-related fibroblasts can promote cancer development and progression by modulating the integrin signaling pathway (41–43), which has also been linked with tumor progression and metastasis (44). Considering that fibroblasts are the main cell types in DC-containing fibrous sarcomas, whether analogous molecular mechanisms exist in this pathology remains to be fully elucidated. In addition, our analysis found that the time to tumor dedifferentiation was closely related to the OS of DC patients. This is easy to understand because a short period of dedifferentiation may likely hint at a highly invasive tumor phenotype, thereby leading to adverse clinical outcomes. In agreement with published reports (17), we also revealed that wide resection of tumors was correlated with a favorable DC prognosis, with surgical treatment as the only independent predictor. These results highlight the importance of wide resection in the treatment of DC patients, which should be achieved whenever possible to prolong patient survival.

Interestingly, we showed that adjuvant chemotherapy negatively affected the PFS of patients with DC. The exact mechanism underlying this condition is unclear. Prior observations have indicated that chemotherapy can cause shedding of the syndecan-1 proteoglycan to accelerate tumor recurrence and progression (45). This finding may provide an explanatory basis for the poor survival of DC patients treated with chemotherapy. Further transcriptome sequencing studies using DC tissue samples before and after chemotherapy may help uncover the precise molecular mechanisms of how chemotherapy promotes DC progression. However, we failed to identify which chemotherapy drugs could lead to a poor DC prognosis because of insufficient information. Future studies with well-documented chemotherapy data are needed to clarify this issue in order to optimize therapy and improve survival. Additionally, this study found that radiotherapy had no significant effect on DC prognosis, similar to previous observations (27, 46). Studies have suggested that radiotherapy may promote DC progression (27). Considering these findings, we recommend that radiotherapy should be arranged with caution for DC patients, although further confirmation is necessary.

### Limitations

Most studies did not provide complete clinicopathological data for PDC/DC patients, which may introduce bias into the results. Despite this, we performed a pooled analysis of individual patient-level data involving DC and PDC subjects from literature to present clinical characteristics of the two chordoma subtypes, which may possibly have implications for clinical decision making of patients. We did this because PDC or DC is a rare entity and collecting sufficient data for analysis in a single institute is difficult. In fact, this method is currently widely used in the literature and is an effective tool for the investigation of rare disease (47). Nevertheless, to reduce the heterogeneities between different studies or between literature data and our local cohort, allow for comparability and then make statistical feasible, we obtained complete and objective patient information (these data cannot have variations among studies, such as age, sex, tumor location, tumor size, etc.), and also simplified criteria for subjective variable grouping in subsequent analysis (divided into low or high and yes or no for most categorical variables). However, additional prospective multicenter cooperative studies with large sample sizes and complete data are still required to confirm our current findings.

## Conclusion

The present study analyzed the clinicopathological characteristics and prognostic factors in a large number of PDC/DC patients and compared their clinical data with that of a CC population. We found that PDC/DC and CC had significantly different clinical features, while PDC and DC shared similarities. In the PDC cohort, adjuvant radiotherapy and chemotherapy were associated with patient survival. In contrast, for DC patients, the presence of a dedifferentiated component, time to tumor dedifferentiation, surgery and radiation therapy significantly affected survival. These data may contribute to a comprehensive understanding of the characteristics of PDC/DC and may be helpful in guiding prognostic risk stratification and therapeutic optimization of patients.

## Data Availability Statement

The original contributions presented in the study are included in the article/**Supplementary Material**. Further inquiries can be directed to the corresponding author.

## Ethics Statement

The study protocol was approved by the Institutional Review Board at The First Affiliated Hospital, University of South China, Hunan, P.R. China. The patients/participants provided their written informed consent to participate in this study.

## Author Contributions

All authors participated in data acquisition. WH, G-HL, JL, and M-XZ contributed to the conception and design of the study. F-SL, B-WZ, WH, and M-XZ did the data analysis and interpretation. T-LZ, WH, Y-GY, and M-XZ contributed to drafting and revision of the manuscript. All authors contributed to the article and approved the submitted version.

## Funding

This work was supported by the National Natural Science Foundation of China (82003802 to T-LZ, 81871821 to JL and 82002364 M-XZ), Natural Science Foundation of Hunan Province (2019JJ50542 to T-LZ), Project for Clinical Research of Hunan Provincial Health Commission (20201978 to T-LZ and 20201956 to M-XZ) and China Scholarship Council (CSC201808430085 to T-LZ).

## Conflict of Interest

The authors declare that the research was conducted in the absence of any commercial or financial relationships that could be construed as a potential conflict of interest.

## Publisher’s Note

All claims expressed in this article are solely those of the authors and do not necessarily represent those of their affiliated organizations, or those of the publisher, the editors and the reviewers. Any product that may be evaluated in this article, or claim that may be made by its manufacturer, is not guaranteed or endorsed by the publisher.
