# Supplementary material for: Clinicopathological and Prognostic Characteristics in Dedifferentiated/Poorly Differentiated Chordomas: A Pooled Analysis of Individual Patient Data From 58 Studies and Comparison With Conventional Chordomas
Source: Front Oncol. 2021 Aug 13;11:686565. doi: 10.3389/fonc.2021.686565 (PMC8418060; doi:10.3389/fonc.2021.686565)
Supplement: Supplementary file 1 [file DataSheet_1.doc]

**Supplemental Digital Content 1.** Detailed information on the antibodies used for the immunohistochemical assay

| Antibody | Host Species | Description | Clone Number | Catalog Number | Company | Dilution |
| --- | --- | --- | --- | --- | --- | --- |
| Brachyury | Mouse | Monoclonal | 1H9A2 | ab140661 | Abcam | 1:200 |
| Pan-cytokeratin | Mouse | Monoclonal | AE1/AE3 | ab27988 | Abcam | 1:20 |
| S-100 | Rabbit | Polyclonal | None | ab76729 | Abcam | 1:100 |
| Vimentin | Rabbit | Polyclonal | None | ab137321 | Abcam | 1:400 |
| MUC1 (EMA) | Rabbit | Monoclonal | EPR1023 | ab109185 | Abcam | 1:500 |
| SMARCB1 (INI-1) | Mouse | Monoclonal | 3-00E-010 | ABIN782370 | ABNOVA | 1:200 |

EMA, epithelial membrane antigen; MUC1, mucin 1; SMARCB1, SWI/SNF related, matrix associated, actin dependent regulator of chromatin, subfamily B, member 1; INI-1, integrase interactor 1.

**
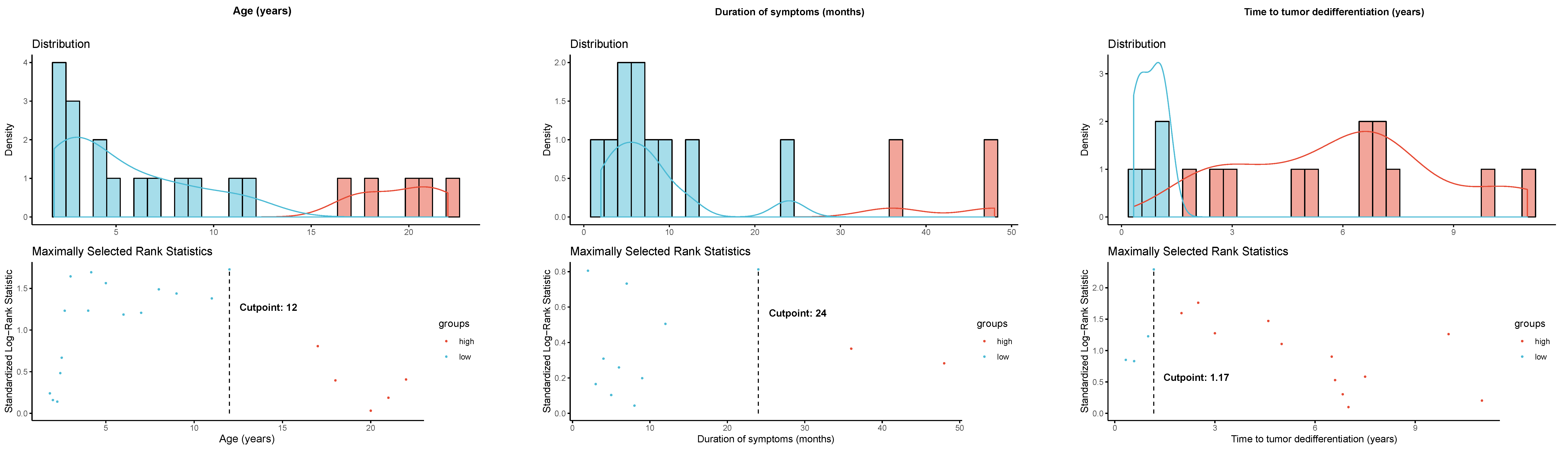
**

**Supplemental Digital Content 2.** Cut-off determination by using maximally selected rank statistics for patient age in PDC cohort (*Left*), as well as duration of symptoms (*Middle*) and time to dedifferentiation in DC cohort (*Right*) in survival analysis, with the overall survival as the outcome parameter. Patients were divided into high-risk and low-risk groups according to the cut-off values. PDC, poorly differentiated chordoma; DC, dedifferentiated chordoma.

**Supplemental Digital Content 3.** List of the included studies addressing patients with poorly differentiated chordoma/dedifferentiated chordoma in the literature.

1. Buccoliero A, Caporalini C, Scagnet M, et al. A Diagnostic Pitfall: Atypical Teratoid Rhabdoid Tumor Versus Dedifferentiated/Poorly Differentiated Chordoma: Analysis of a Mono-institutional Series. *Applied immunohistochemistry & molecular morphology*. 2019;27(2): 147-54.

2. Kaneko Y, Sato Y, Iwaki T, Shin R, Tateishi J, Fukui M. Chordoma in early childhood: a clinicopathological study. *Neurosurgery*. 1991;29(3): 442-6.

3. Shih A, Cote G, Chebib I, et al. Clinicopathologic characteristics of poorly differentiated chordoma. *Modern pathology*. 2018;31(8): 1237-45.

4. Rekhi B, Banerjee D, Ramadwar M, Bajpai J, Jambhekar N. Clinicopathologic features of four rare types of chordomas, confirmed by brachyury immunostaining. *Indian journal of pathology & microbiology*. 2017;60(3): 350-4.

5. Mobley B, McKenney J, Bangs C, et al. Loss of SMARCB1/INI1 expression in poorly differentiated chordomas. *Acta neuropathologica*. 2010;120(6): 745-53.

6. Renard C, Pissaloux D, Decouvelaere A, Bourdeaut F, Ranchère D. Non-rhabdoid pediatric SMARCB1-deficient tumors: overlap between chordomas and malignant rhabdoid tumors? *Cancer genetics*. 2014;207(9): 384-9.

7. Cha Y, Hong C, Kim D, Lee S, Park H, Kim S. Poorly differentiated chordoma with loss of SMARCB1/INI1 expression in pediatric patients: A report of two cases and review of the literature. *Neuropathology*. 2018;38(1): 47-53.

8. Chavez J, Nasir UD, Memon A, Perry A. Anaplastic chordoma with loss of INI1 and brachyury expression in a 2-year-old girl. *Clinical neuropathology*. 2014;33(6): 418-20.

9. Yadav R, Sharma M, Malgulwar P, et al. Prognostic value of MIB-1, p53, epidermal growth factor receptor, and INI1 in childhood chordomas. *Neuro-oncology*. 2014;16(3): 372-81.

10. Antonelli M, Raso A, Mascelli S, et al. SMARCB1/INI1 Involvement in Pediatric Chordoma: A Mutational and Immunohistochemical Analysis. *The American journal of surgical pathology*. 2017;41(1): 56-61.

11. Rekhi B, Kosemehmetoglu K, Rane S, Soylemezoglu F, Bulut E. Poorly Differentiated Chordomas Showing Loss of INI1/SMARCB1: A Report of 2 Rare Cases With Diagnostic Implications. *International journal of surgical pathology*. 2018;26(7): 637-43.

12. Owosho A, Zhang L, Rosenblum M, Antonescu C. High sensitivity of FISH analysis in detecting homozygous SMARCB1 deletions in poorly differentiated chordoma: a clinicopathologic and molecular study of nine cases. *Genes, chromosomes & cancer*. 2018;57(2): 89-95.

13. Mavrogenis A, Angelini A, Panagopoulos G, et al. Aggressive Chordomas: Clinical Outcome of 13 Patients. *Orthopedics* 2017;40(2): e248-e54.

14. Smith J, Reuter V, Demas B. Case report 576. Anaplastic sacrococcygeal chordoma (dedifferentiated chordoma). *Skeletal radiology*. 1989;18(7): 561-4.

15. Ridenour R, Ahrens W, Folpe A, Miller D. Clinical and histopathologic features of chordomas in children and young adults. *Pediatric and developmental pathology*. 2010;13(1): 9-17.

16. Makhdoomi R, Ramzan A, Khursheed N, et al. Clinicopathological characteristics of chordoma: an institutional experience and a review of the literature. *Turkish neurosurgery*. 2013;23(6): 700-6.

17. Bandyopadhyay A, Goswami B, Pramanik R, Majumdar K, Gangopadhyay M. Cytopathological dilemma of anaplastic sacral chordoma with radiological and histological corroboration. *Turk patoloji dergisi*. 2011;27(2): 157-60.

18. Chou W, Hung Y, Lu C, Yeh K, Sheu S, Liaw C. De novo dedifferentiated chordoma of the sacrum: a case report and review of the literature. *Chang Gung medical journal*. 2009;32(3): 330-5.

19. Meis J, Raymond A, Evans H, Charles R, Giraldo A. "Dedifferentiated" chordoma. A clinicopathologic and immunohistochemical study of three cases. *The American journal of surgical pathology*. 1987;11(7): 516-25.

20. Fleming G, Heimann P, Stephens J, et al. Dedifferentiated chordoma. Response to aggressive chemotherapy in two cases. *Cancer*. 1993;72(3): 714-8.

21. Layfield L, Liu K, Dodd L, Olatidoye B. "Dedifferentiated" chordoma: a case report of the cytomorphologic findings on fine-needle aspiration. *Diagnostic cytopathology*. 1998;19(5): 378-81.

22. Munshi H, Merajver S, Valdez R, Baker L, Cooney K. Metastatic dedifferentiated chordoma with elevated beta-hCG: a case report. *American journal of clinical oncology*. 2002;25(3): 274-6.

23. Makek M, Leu H. Malignant fibrous histiocytoma arising in a recurrent chordoma. Case report and electron microscopic findings. *Virchows Arch A Pathol Anat Histol*. 1982;397(3): 241-50.

24. Miettinen M, Lehto V, Virtanen I. Malignant fibrous histiocytoma within a recurrent chordoma. A light microscopic, electron microscopic, and immunohistochemical study. *American journal of clinical pathology*. 1984;82(6): 738-43.

25. Hruban R, May M, Marcove R, Huvos A. Lumbo-sacral chordoma with high-grade malignant cartilaginous and spindle cell components. *The American journal of surgical pathology*. 1990;14(4): 384-9.

26. Saito A, Hasegawa T, Shimoda T, et al. Dedifferentiated chordoma: a case report. *Japanese journal of clinical oncology*. 1998;28(12): 766-71.

27. Hanna S, Tirabosco R, Amin A, et al. Dedifferentiated chordoma: a report of four cases arising 'de novo'. *The Journal of bone and joint surgery. British volume*. 2008;90(5): 652-6.

28. Bisceglia M, D'Angelo V, Guglielmi G, Dor D, Pasquinelli G. Dedifferentiated chordoma of the thoracic spine with rhabdomyosarcomatous differentiation. Report of a case and review of the literature. *Annals of diagnostic pathology*. 2007;11(4): 262-73.

29. Masood Q, Bilal M, Tariq A, Khan S, Qureshi A. Dedifferentiated chordoma with a sarcomatous component: an overlooked diagnosis. *Journal of Ayub Medical College, Abbottabad*. 2009;21(1): 164-5.

30. Chan A, Tsang W, Chan G, Lam Y, Chan M. Dedifferentiated chordoma with rhabdomyoblastic differentiation. *Pathology*. 2007;39(2): 277-80.

31. Gil Z, Fliss D, Voskoboinik N, et al. Cytogenetic analysis of three variants of clival chordoma. *Cancer genetics and cytogenetics*. 2004;154(2): 124-30.

32. Kayani B, Sewell M, Hanna S, et al. Prognostic factors in the operative management of dedifferentiated sacral chordomas. *Neurosurgery*. 2014;75(3): 269-75; discussion 75.

33. Kim J, Lee J, Koh J, Park M, Chang U. Establishment and characterization of a chordoma cell line from the tissue of a patient with dedifferentiated-type chordoma. *Journal of neurosurgery. Spine*. 2016;25(5): 626-35.

34. Kearns C, Kearns C. Fifty-four-month survival in a 3-year-old child presenting with an aggressive metastatic dedifferentiated clival chordoma. *BMJ case reports*. 2016;2016:bcr2016216017.

35. Frankl J, Grotepas C, Stea B, Lemole G, Chiu A, Khan R. Chordoma dedifferentiation after proton beam therapy: a case report and review of the literature. *Journal of medical case reports*. 2016;10(1): 280.

36. Dhall G, Traverso M, Finlay J, Shane L, Gonzalez-Gomez I, Jubran R. The role of chemotherapy in pediatric clival chordomas. *Journal of neuro-oncology*. 2011;103(3): 657-62.

37. Choi Y, Kim T. Malignant fibrous histiocytoma in chordoma--immunohistochemical evidence of transformation from chordoma to malignant fibrous histiocytoma. *Yonsei medical journal*. 1994;35(2): 239-43.

38. Tsitouras V, Wang S, Dirks P, et al. Management and outcome of chordomas in the pediatric population: The Hospital for Sick Children experience and review of the literature. *Journal of clinical neuroscience*. 2016;34: 169-76.

39. Jambhekar N, Rekhi B, Thorat K, Dikshit R, Agrawal M, Puri A. Revisiting chordoma with brachyury, a "new age" marker: analysis of a validation study on 51 cases. *Archives of pathology & laboratory medicine*. 2010;134(8): 1181-7.

40. Kato S, Gasbarrini A, Ghermandi R, Gambarotti M, Bandiera S. Spinal chordomas dedifferentiated to osteosarcoma: a report of two cases and a literature review. *European spine journal*. 2016;25 Suppl 1:251-6.

41. Barrenechea I, Perin N, Triana A, Lesser J, Costantino P, Sen C. Surgical management of chordomas of the cervical spine. *Journal of neurosurgery. Spine*. 2007;6(5): 398-406.

42. Kim S, Cho W, Chang U, Youn S. Two Cases of Dedifferentiated Chordoma in the Sacrum. *Korean Journal of Spine*. 2015;12(3): 230-4.

43. Morimitsu Y, Aoki T, Yokoyama K, Hashimoto H. Sarcomatoid chordoma: chordoma with a massive malignant spindle-cell component. *Skeletal radiology*. 2000;29(12): 721-5.

44. Hruban R, Traganos F, Reuter V, Huvos A. Chordomas with malignant spindle cell components. A DNA flow cytometric and immunohistochemical study with histogenetic implications. *The American journal of pathology*. 1990;137(2): 435-47.

45. Rutkowski M, Birk H, Wood M, et al. Metastatic clival chordoma: a case report of multiple extraneural metastases following resection and proton beam radiotherapy in a 5-year old boy. *Journal of neurosurgery. Pediatrics*. 2017;19(5): 531-7.

46. Knechtges T. Sacrococcygeal chordoma with sarcomatous features (spindle cell metaplasia). *American journal of clinical pathology*. 1970;53(5): 612-6.

47. Halpern J, Kopolovic J, Catane R. Malignant fibrous histiocytoma developing in irradiated sacral chordoma. *Cancer*. 1984;53(12): 2661-2.

48. Belza M, Urich H. Chordoma and malignant fibrous histiocytoma. Evidence for transformation. *Cancer*. 1986;58(5): 1082-7.

49. Gounder M, Zhu G, Roshal L, et al. Immunologic Correlates of the Abscopal Effect in a SMARCB1/INI1-negative Poorly Differentiated Chordoma after EZH2 Inhibition and Radiotherapy. *Clinical cancer research*. 2019;25(7): 2064-71.

50. Nachwalter R, Rothrock R, Katsoulakis E, et al. Treatment of dedifferentiated chordoma: a retrospective study from a large volume cancer center. *Journal of neuro-oncology*. 2019;144(2): 369-76.

51. Asioli S, Zoli M, Guaraldi F, et al. Peculiar pathological, radiological and clinical features of skull-base de-differentiated chordomas. Results from a referral centre case-series and literature review. *Histopathology*. 2020;76(5): 731-9.

52. Hara T, Kawahara N, Tsuboi K, Shibahara J, Ushiku T, Kirino T. Sarcomatous transformation of clival chordoma after charged-particle radiotherapy. Report of two cases. *Journal of neurosurgery*. 2006;105(1): 136-41.

53. Ng W, Tang V. Crush preparation findings of "sarcomatoid" chordoma of the sacrum: report of a case with histologic, immunohistochemical, and ultrastructural correlation. *Diagnostic cytopathology*. 2001;25(6): 406-10.

54. Miettinen M, Karaharju E, Järvinen H. Chordoma with a massive spindle-cell sarcomatous transformation. A light- and electron-microscopic and immunohistological study. *The American journal of surgical pathology*. 1987;11(7): 563-70.

55. Volpe R, Mazabraud A. A clinicopathologic review of 25 cases of chordoma (a pleomorphic and metastasizing neoplasm). *The American journal of surgical pathology*. 1983;7(2): 161-70.

56. Nanda A, Hirsh L, Antoiniades K. Malignant fibrous histiocytoma in a recurrent thoracic chordoma: case report and literature review. *Neurosurgery*. 1991;28(4): 588-92.

57. Gong L, Liu W, Ding Y, Sun X, Zhang M, Huang X. [Dedifferentiated chordoma of sacrococcygeal region: a clinicopathologic analysis and review of literature]. *Zhonghua bing li xue za zhi = Chinese journal of pathology*. 2018;47(5): 349-53.

58. Feng J, Chen L, Ma Y, Yang H, Chen L. [Poorly-differentiated chordoma with INI1 loss: a clinicopathologic study]. *Zhonghua bing li xue za zhi = Chinese journal of pathology*. 2017;46(10): 695-8.


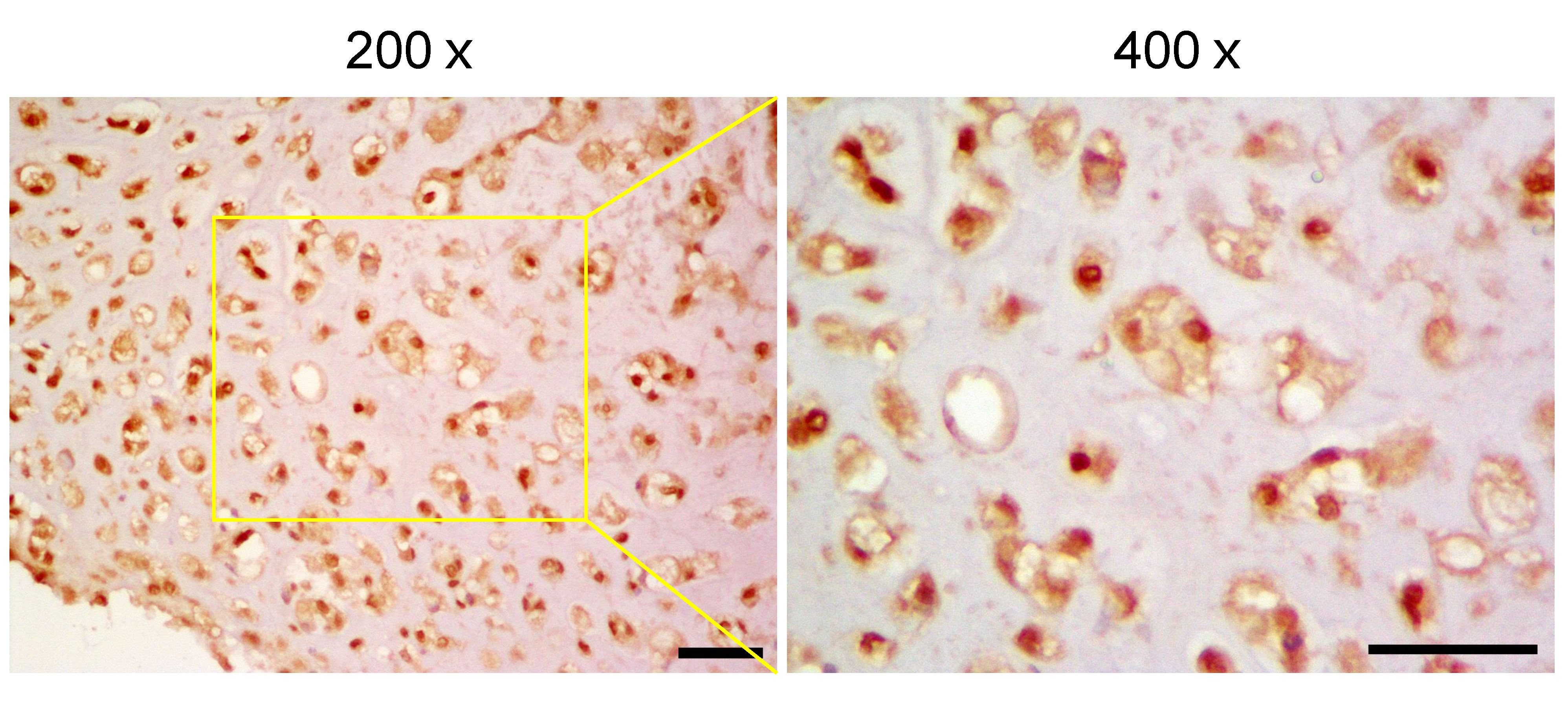


**Supplemental Digital Content 4.** Representative immunostaining images showing positive nuclear INI-1 expression in classic chordoma tissues. INI-1, integrase interactor 1. Scale bar = 100 μm.


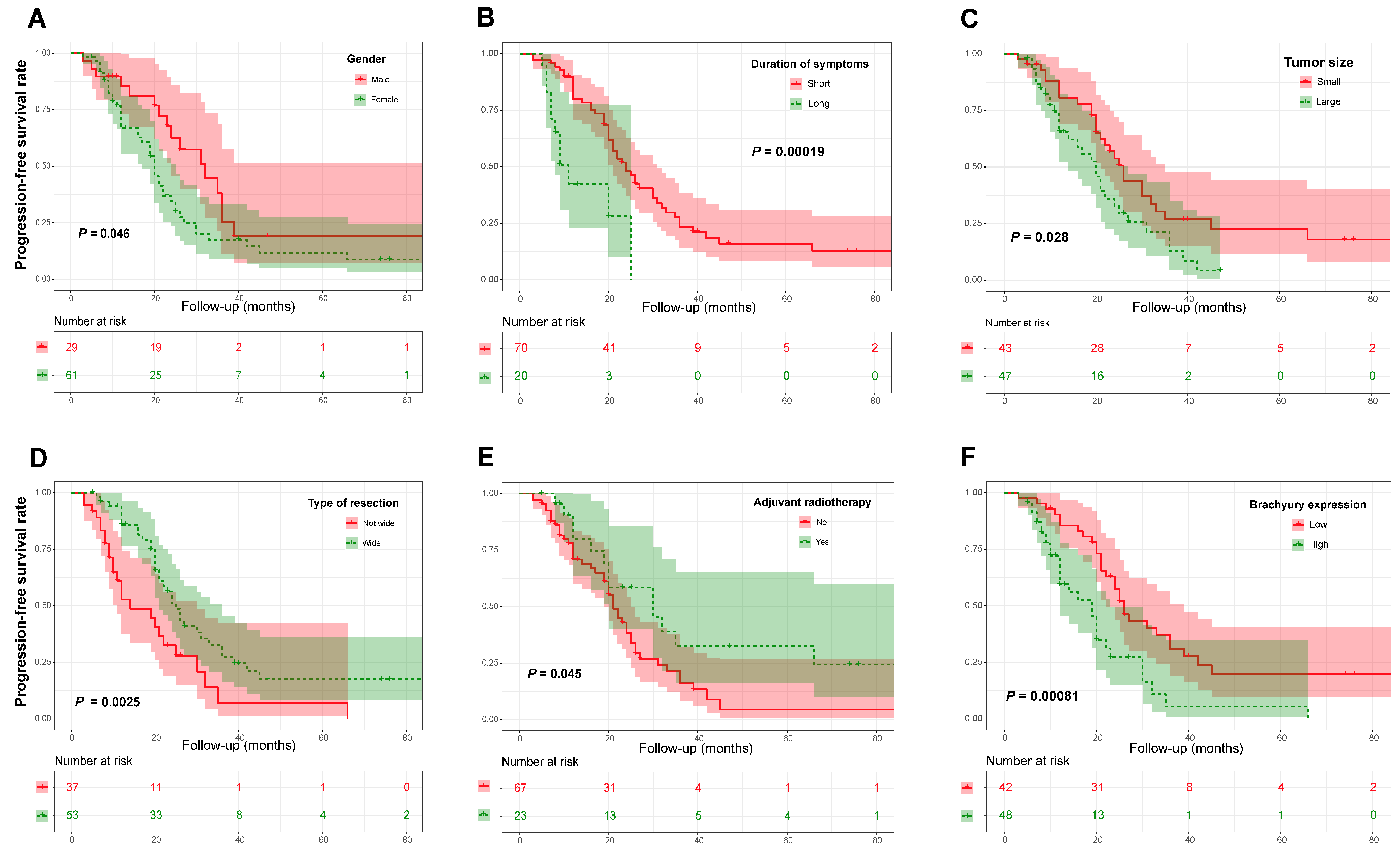


**Supplemental Digital Content 5.** Kaplan-Meier curves of progression-free survival of patients with classic chordoma stratified by gender (**A**), duration of symptoms (**B**), tumor size (**C**), resection type (**D**), adjuvant radiotherapy (**E**) and Brachyury expression (**F**).

**Supplemental Digital Content 6.** Univariate analyses of prognostic factors for progression-free survival and overall survival in patients with classic chordoma^a^

| Factors | Categories | progression-free survival | | | | overall survival | | | |
| --- | --- | --- | --- | --- | --- | --- | --- | --- | --- |
|  |  | n | Median survival (months) | χ^2^ | *P*-value | n | Median survival (months) | χ^2^ | *P*-value |
| Age (years) | ≤ 50 | 37 | 24 | 1.954 | 0.162 | 37 | 97 | 1.986 | 0.159 |
|  | > 50 | 53 | 21 |  |  | 53 | 35 |  |  |
| Gender | Female | 61 | 20 | 3.970 | **0.046** | 61 | 35 | 2.583 | 0.108 |
|  | Male | 29 | 32 |  |  | 29 | 97 |  |  |
| Duration of symptoms (months) | ≤ 36.5 | 70 | 24 | 13.928 | < **0.001** | 70 | 84 | 18.353 | < **0.001** |
|  | > 36.5 | 20 | 11 |  |  | 20 | 23 |  |  |
| Tumor size (largest diameter, cm) | ≤ 5 | 43 | 26 | 4.806 | **0.028** | 43 | 84 | 1.765 | 0.184 |
|  | > 5 | 47 | 20 |  |  | 47 | 48 |  |  |
| Tumor location | Spine | 77 | 22 | 2.693 | 0.101 | 77 | 56 | 1.061 | 0.303 |
|  | Skull base | 13 | 30 |  |  | 13 | 84 |  |  |
| Type of resection | Not wide | 37 | 14 | 9.169 | **0.002** | 37 | 28 | 10.302 | **0.001** |
|  | Wide | 53 | 25 |  |  | 53 | 97 |  |  |
| Adjuvant radiotherapy | No | 67 | 21 | 4.008 | **0.045** | 67 | 48 | 0.847 | 0.357 |
|  | Yes | 23 | 30 |  |  | 23 | 97 |  |  |
| Brachyury | Low | 42 | 26 | 11.211 | **0.001** | 42 | 128 | 15.614 | < **0.001** |
|  | High | 48 | 19 |  |  | 48 | 27 |  |  |
| CK expression | Low | 66 | 24 | 0.025 | 0.874 | 66 | 73 | 0.332 | 0.564 |
|  | High | 24 | 20 |  |  | 24 | 35 |  |  |
| S-100 expression | Low | 67 | 22 | 0.486 | 0.486 | 67 | 73 | 0.055 | 0.815 |
|  | High | 23 | 25 |  |  | 23 | 48 |  |  |
| EMA expression | Low | 68 | 22 | 0.197 | 0.657 | 68 | 73 | 0.035 | 0.852 |
|  | High | 22 | 25 |  |  | 22 | 56 |  |  |
| Vimentin expression | Low | 69 | 22 | 0.038 | 0.845 | 69 | 57 | 0.577 | 0.447 |
|  | High | 21 | 21 |  |  | 21 | - |  |  |

Bold values indicate *P* < 0.05; CK, pancytokeratin; EMA, epithelial membrane antigen; ^a^analysis was not performed for tumoral INI-1 expression and patient outcomes as all cases had positive INI-1 staining in the classic chordoma group; INI-1, integrase interactor 1.


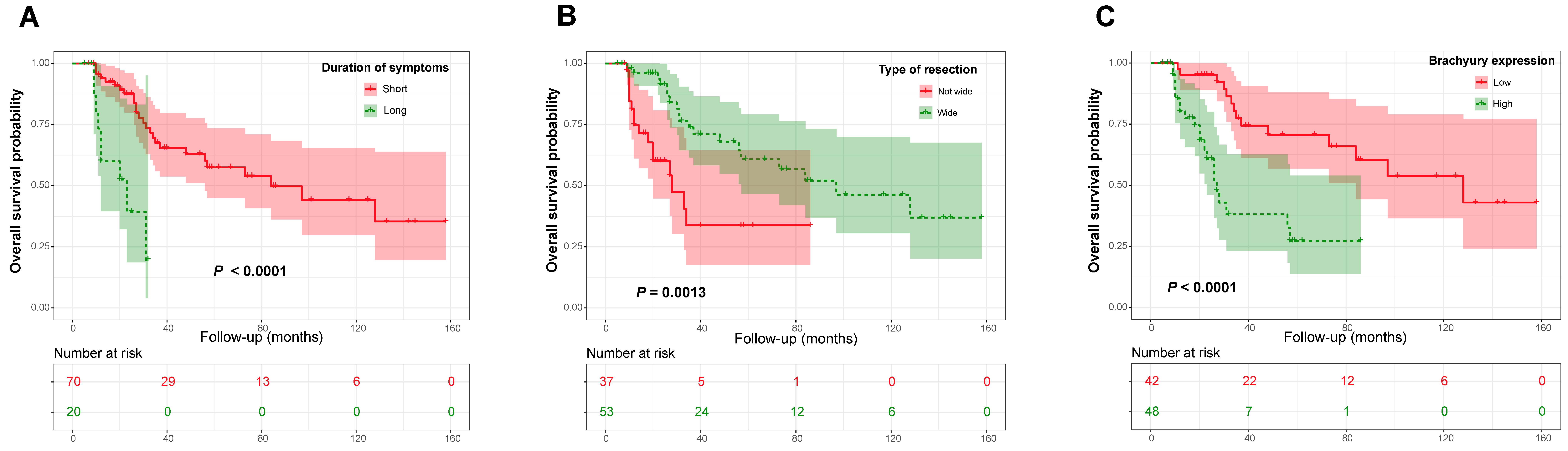


**Supplemental Digital Content 7.** Kaplan-Meier curves of overall survival of patients with classic chordoma stratified by duration of symptoms (**A**), resection type (**B**) and Brachyury expression (**C**).

**Supplemental Digital Content 8.** Multivariate Cox proportional hazard analyses of prognostic factors for progression-free survival and overall survival in patients with classic chordoma

| Factors | Categories | progression-free survival | | Factors | Categories | overall survival | |
| --- | --- | --- | --- | --- | --- | --- | --- |
|  |  | *P*-value | HR (95% CI) |  |  | *P*-value | HR (95% CI) |
| Gender | Female/Male | **0.038** | 1.909 (1.035-3.520) | Duration of symptoms (months) | > 36.5/≤ 36.5 | **0.006** | 1.023 (1.007-1.040) |
| Duration of symptoms (months) | > 36.5/≤ 36.5 | 0.561 | 1.005 (0.989-1.021) | Type of resection | Wide/Not wide | **0.030** | 0.439 (0.209-0.922) |
| Tumor size | > 5 cm/≤ 5 cm | 0.027 | 1.094 (1.010-1.184) | Brachyury expression | Low/High | **0.040** | 0.427 (0.190-0.961) |
| Type of resection | Wide/Not wide | **0.002** | 0.378 (0.202-0.709) |  |  |  |  |
| Adjuvant radiotherapy | No/Yes | < **0.001** | 5.003 (2.137-11.715) |  |  |  |  |

Bold values indicate *P* < 0.05.
